# A multicomponent intervention for the management of chronic pain in older adults: study protocol for a randomized controlled trial

**DOI:** 10.1186/s13063-017-2270-3

**Published:** 2017-11-09

**Authors:** Sheung-Tak Cheng, Ka Long Chan, Rosanna W. L. Lam, Monique H. T. Mok, Phoon Ping Chen, Yu Fat Chow, Joanne W. Y. Chung, Alexander C. B. Law, Jenny S. W. Lee, Edward M. F. Leung, Cindy W. C. Tam

**Affiliations:** 1 Department of Health and Physical Education, The Education University of Hong Kong, 10 Lo Ping Road, Tai Po, New Territories Hong Kong; 2 Department of Clinical Psychology, Norwich Medical School, University of East Anglia, Norfolk, NR4 7TJ UK; 3 Department of Rehabilitation and Extended Care, Kowloon Hospital, 147A Argyle Street, Kowloon, Hong Kong; 4 Department of Anesthesiology & Operating Services, Alice Ho Miu Ling Nethersole Hospital, 11 Chuen On Road, Tai Po, New Territories Hong Kong; 5 Department of Anesthesiology & Operating Theatre Services, Queen Elizabeth Hospital, 30 Gascoigne Road, Kowloon, Hong Kong; 6 Department of Medicine and Geriatrics, Princess Margaret Hospital, 2-10 Princess Margaret Hospital Road, Lai Chi Kok, Kowloon, Hong Kong; 7 Department of Medicine, Alice Ho Miu Ling Nethersole Hospital, 11 Chuen On Road, Tai Po, New Territories Hong Kong; 8 Department of Medicine and Geriatrics, United Christian Hospital, 130 Hip Wo Street, Kwun Tong, Kowloon, Hong Kong; 9 Department of Psychiatry, North District Hospital, 9 Po Kin Road, Sheung Shui, New Territories Hong Kong

**Keywords:** Chronic pain management, Physical exercise, Cognitive behavioral techniques

## Abstract

**Background:**

Studies have shown that physical interventions and psychological methods based on the cognitive behavioral approach are efficacious in alleviating pain and that combining both tends to yield more benefits than either intervention alone. In view of the aging population with chronic pain and the lack of evidence-based pain management programs locally, we developed a multicomponent intervention incorporating physical exercise and cognitive behavioral techniques and examined its long-term effects against treatment as usual (i.e., pain education) in older adults with chronic musculoskeletal pain in Hong Kong.

**Methods/design:**

We are conducting a double-blind, cluster-randomized controlled trial. A sample of 160 participants aged ≥ 60 years will be recruited from social centers or outpatient clinics and will be randomized on the basis of center/clinic to either the multicomponent intervention or the pain education program. Both interventions consist of ten weekly sessions of 90 minutes each. The primary outcome is pain intensity, and the secondary outcomes include pain interference, pain persistence, pain self-efficacy, pain coping, pain catastrophizing cognitions, health-related quality of life, depressive symptoms, and hip and knee muscle strength. All outcome measures will be collected at baseline, postintervention, and at 3 and 6 months follow-up. Intention-to-treat analysis will be performed using mixed-effects regression to see whether the multicomponent intervention alleviates pain intensity and associated outcomes over and above the effects of pain education (i.e., a treatment × time intervention effect).

**Discussion:**

Because the activities included in the multicomponent intervention were carefully selected for ready implementation by allied health professionals in general, the results of this study, if positive, will make available an efficacious, nonpharmacological pain management program that can be widely adopted in clinical and social service settings and will hence improve older people’s access to pain management services.

**Trial registration:**

Chinese Clinical Trial Registry, ChiCTR-IIR-16008387. Registered on 28 April 2016.

## Background

Pain is a common and debilitating problem among older people, affecting 50% or more of community-dwelling older adults and up to 80% of nursing home residents [1]. With rapidly aging populations, the number of people worldwide experiencing pain is expected to increase sharply in the decades to come. Research in Western countries consistently shows the prevalence of pain rising to a peak of 30–65% in the age group of 55–65 years and then declining somewhat to around 25–55% among those aged 85 years or over [1–3]. Worldwide, the most common pain disorders reported by older people include arthritis (e.g., rheumatoid arthritis, osteoarthritis), back pain, and fibromyalgia [4, 5]. The high prevalence of pain in the older population can be explained by the higher rates of surgical procedures, injury, and disease in later life. Age-related neurophysiological changes, such as decline in pain tolerance and increased pain sensitivity, also contribute to an increased risk of pain in older adults [6–8]. Even if there is a slight reduction in pain prevalence in the very advanced age group due to the survivor effect (i.e., persons with less favorable health conditions would have had early mortality), it is estimated that 25–55% of very old adults have at least one pain problem [9].

**Fig. 1:**
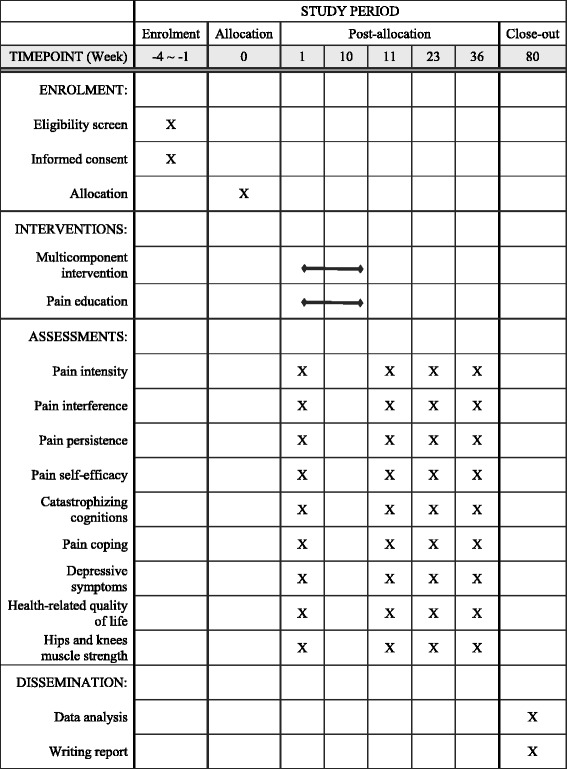
Standard Protocol Items: Recommendations for Interventional Trials (SPIRIT) figure

Despite the high prevalence of pain and its medical and psychiatric comorbidities in later life, pain is often underreported in older people. Many older persons are stoic regarding mild aches and pain sensations because it is often assumed that pain is an inevitable part of the aging process and should be tolerated. In fact, there are common cultural and social myths about pain and its management in older people that are shared by healthcare professionals as well as the general public, who often underestimate the extent of pain experienced by older people [10, 11]. For example, many believe that older people get used to the pain and should therefore be given a lower priority for treatment than younger patients with similar clinical characteristics [12, 13]. In addition, many elders assume a passive role in healthcare and are hesitant to report pain [14, 15]. As a result, older people may be at a disadvantage in terms of access to pain management services, such as being underrepresented in multidisciplinary pain management programs [13]. Even after they are admitted to such programs, older people are often provided with less treatment and fewer treatment options [16, 17]. This stands in contrast to the fact that the pain they experience is often of a chronic nature and has potentially serious physical and psychosocial consequences, including a lower health-related quality of life, increased depression and anxiety, sleep disturbances, social isolation, and impaired cognitive performance and functional ability [18–21]. For instance, a study showed that 46% and 53% of chronic nonmalignant pain patients aged ≥ 72 years were diagnosed with major depressive disorder and reported suicidal ideation, respectively [22].

Although it has been widely recognized that multidisciplinary pain management that incorporates analgesic medication with nonpharmacological approaches such as psychological interventions (e.g., cognitive behavioral therapy), physiotherapy, occupational therapy, and alternative medicine is more effective in pain reduction [23–25], the pharmacological approach remains the mainstay treatment for pain relief among older patients in many parts of the world, including Hong Kong. Part of the reason is the lack of evidence-based nonpharmacological pain management programs in these communities.

In the pain management literature, both physical activity and cognitive behavioral therapy have been found to be effective in alleviating pain intensity and the degree to which pain interferes with daily functioning in adults in general [23, 24] and older adults in particular [25], leading to one evidence-based guideline for combining both approaches in the management of chronic low back pain [26]. However, whereas there were relatively more physical activity interventions for older people, only a few studies have incorporated cognitive behavioral techniques, and most of these studies suffer from small sample sizes and no or short follow-up [25, 27]. Nevertheless, it is noteworthy that a recent randomized trial of 141 older adults with chronic pain showed that cognitive behavioral techniques, when combined with physical exercise, were superior to exercise alone or a wait-list control group across a range of outcomes, including pain intensity, pain-related distress and disability, dysfunctional cognitions, and a functional reach test, with small to medium effect sizes 1 month postintervention [28]. This was, to the best of our knowledge, the only study to date incorporating cognitive behavioral techniques and physical exercise in a randomized trial for older adults with chronic pain. More studies are necessary to verify the therapeutic benefits of such multicomponent interventions and to see if the benefits can be generalized to other cultural groups. We therefore are conducting a cluster-randomized controlled trial to investigate the long-term effects (up to 6 months postintervention) of a multimodal intervention combining physical exercise and cognitive behavioral techniques on alleviating the intensity of pain in older adults with chronic musculoskeletal pain in Hong Kong.

## Methods/design

### Study objectives and design

In a cluster-randomized controlled trial with two arms, we aim to evaluate the long-term effects of a newly developed multicomponent pain management intervention incorporating physical exercise and cognitive behavioral techniques against a pain education program (i.e., treatment as usual) in older adults. The activities included in the multicomponent intervention were carefully selected so that they could be readily implementable by allied health professionals in general to enhance adoption of the program in clinical practice, especially in places such as Hong Kong, where personnel in certain specialties are lacking. The primary outcome is pain intensity, whereas the secondary outcomes include pain interference, pain persistence, pain self-efficacy, pain coping, pain catastrophizing cognitions, health-related quality of life, depressive symptoms, and hip and knee muscle strength. All outcome measures will be collected at baseline, postintervention, and 3 and 6 months follow-up. Key aspects of the study design can be found in Fig. 1.

### Interventions

Both interventions have ten weekly sessions of 90 minutes each. The pain education program was derived from a brochure on pain management that is distributed in public hospitals and clinics in Hong Kong (i.e., treatment as usual), and it covers general information about chronic pain (including the impact, cause, and type), pharmacotherapy and nonpharmacological pain relief strategies, community resources, and pain-related sleep difficulties and possible ways to alleviate them [29]. It was necessary to adapt the educational brochure, a reading material, into an interactive format with the same duration as the multicomponent intervention program so that any difference in treatment outcome between the two programs cannot be attributed to differences in format, exposure, and intensity (i.e., placebo effects). *See* Table 1 for details of the pain education program.

**Table 1:** Pain education intervention

Session	Content
Session 1	Situation of chronic pain among Hong Kong older adults Difference between chronic pain and acute pain Types of chronic pain
Session 2	Review of previous session Impact of chronic pain on older adults Interaction between chronic pain, bodily function, and psychological condition Impact of chronic pain on social relationships with others Understanding the vicious cycle related to chronic pain
Session 3	Review of previous session Causes of chronic pain Different types of chronic pain
Session 4	Review of previous session Addressing misunderstandings about chronic pain Drug and nondrug therapies
Session 5	Review of previous session Introducing the pain ladder recommended by the World Health Organization How to take medicine appropriately Addressing the concerns of taking medicine Side effects of drugs and things to notice (part A)
Session 6	Review of previous session Side effects of drugs and things to notice (part B) Physiotherapy Stimulation (electrotherapy, acupuncture) Surgery Aromatherapy
Session 7	Review of previous session Psychological strategies to deal with chronic pain Hydrotherapy Music therapy
Session 8	Review of previous session Different types of medical imaging in managing chronic pain How to cooperate with doctors in dealing with pain
Session 9	Review of previous session Adjustment of walking speed Introducing correct bodily postures Introducing safety guidelines for physical exercises How to exercise correctly
Session 10	Review of previous session Sleep problems related to chronic pain Impact of insomnia Managing sleep problems Healthy sleeping habits Social resources

The physical exercise component of the multicomponent intervention was developed on the basis of existing self-help physical exercise manuals [30, 31] and guidelines [32, 33] on exercises suitable for older adults with musculoskeletal pain. The activities used in the intervention include stretching and strengthening exercises that have been shown to be the most effective among different exercise types for alleviating pain and improving function [34]. In addition, participants are given balance training that, along with strengthening exercise, can reduce fall rates in older people by improving the ability to control and maintain the body’s position [32, 35]. Each of the ten sessions begins with a 45-minute physical exercise program that includes a 5-minute warmup and 10-minute stretching, 15-minute strengthening, 10-minute balance, and 5-minute cooldown exercises.

The physical exercises are followed by a 40-minute training on cognitive behavioral techniques before a 5-minute conclusion. The psychological modules of the multicomponent intervention were designed using cognitive behavioral principles, focusing on participants’ cognitions, feelings, and behaviors in response to pain. In particular, the intervention emphasizes motivating participants to modify their dysfunctional thoughts that prevent them from engaging in physical and social activities and exploring realistic alternatives. These modules therefore complement the physical exercise component by reducing resistance to engage in physical and other activities that have the potential to reduce pain and related problems. The other techniques, including relaxation exercise, goal setting, assertive communication skills, self-compassion, positive self-statement, and stress management, are also applied. The modules are based partly on existing manuals on pain management [36–38] and guidelines for pain management for older adults using cognitive behavioral principles [39]. Each session is modeled on the same format, including mood checking, review of previous session, homework review, session objectives, discussion, skills practice, and homework/goal setting. Details of the multicomponent intervention can be found in Table 2 [37–40].

**Table 2:** Multicomponent intervention

Session	Physical exercise	Cognitive behavioral technique
Session 1	Principles and safety guidelines for physical exercises Warming up Neck stretching Shoulder stretching Arm strengthening Wall push-up Balance exercises Cooling down	Participants’ expectation of the course and personal goals Discussion: daily difficulties due to pain Understanding of pain: pain types, theories, and factors that contribute to pain and the consequences Pain management: healthy lifestyle and discussion Homework: pain diary (mood monitor)
Session 2	Warming up Neck stretching Shoulder stretching Arm strengthening Wall push-up Balance exercises Cooling down	Review of homework from previous session Discussion: reflection on effectiveness of physical exercise on dealing with chronic pain Understanding of the stress-pain-appraisal connection Introducing the maintenance cycle: relationships between thought, emotion, behavior, and bodily sensation Homework: pain and stress relationship worksheet
Session 3	Warming up Upper body stretching Back stretching Arm strengthening Balance exercises Cooling down	Review of homework from previous session Discussion: reflection on effectiveness of physical exercise on dealing with chronic pain Review of the maintenance cycle Discussion: how do your thoughts, emotions, and behaviors impact your chronic pain? Identify automatic thoughts and beliefs Introducing unhelpful thinking habits Challenging thoughts Practice the thought record sheet Homework: thought record sheet
Session 4	Warming up Upper body stretching Back stretching Arm strengthening Balance exercises Cooling down	Review of homework from previous session Discussion: reflection on effectiveness of physical exercise on dealing with chronic pain Acceptance of pain Self-statement exercise Homework: self-statement record sheet
Session 5	Warming up Back leg raise Side leg raise Balance exercises Cooling down	Review of homework from previous session Discussion: reflection on effectiveness of physical exercise on dealing with chronic pain Mood monitoring Strategies to cope with stress Breathing exercise Homework: mood thermometer worksheet
Session 6	Warming up Back leg raise Side leg raise Balance exercises Cooling down	Review of homework from previous session Discussion: reflection on effectiveness of physical exercise on dealing with chronic pain Use of public resources Social support Communication skills Muscle relaxation exercise Homework: assertiveness worksheet
Session 7	Warming up Hamstring stretching Knee extension Heel raise Body weight squat Balance exercises Cooling down	Review of homework from previous session Discussion: reflection on effectiveness of physical exercise on dealing with chronic pain Case discussion Activity scheduling The importance of sleep Managing sleep problems Homework: activity diary
Session 8	Warming up Hamstring stretching Knee extension Heel raise Body weight squat Balance exercises Cooling down	Review of homework from previous session Discussion: reflection on effectiveness of physical exercise on dealing with chronic pain Case discussion: taking good care of yourself Identifying personal strengths by reflecting on past successful coping efforts Rewarding yourself Grounding techniques Homework: keeping a log of thankful events and grounding exercise
Session 9	Warming up Neck stretching Shoulder stretching Upper body stretching Back stretching Arm strengthening Wall push-up Balance exercises Cooling down	Review of homework from previous session Discussion: reflection on effectiveness of physical exercise on dealing with chronic pain Discussion: what do you want to do but have to give up because of the pain, and what else can you do despite pain? Goal setting Homework: goal setting worksheet (SMART [specific, measurable, attainable, relevant, time-bound] goals)
Session 10	Warming up Hamstring stretching Knee stretching Back leg raise Side leg raise Body weight squat Balance exercises Cooling down Hot and cold compress	Review of homework from previous session Discussion: reflection on effectiveness of physical exercise on dealing with chronic pain Reviewing concepts and skills learned in the course thus far Exercise: relapse prevention and reviewing coping strategies by using worksheet Take-home messages

Interventions will be conducted in a quiet room in the setting from which the participants were recruited. Each group will consist of 6–12 participants. To ensure consistency in delivery, one research assistant with a master’s degree in applied psychology will deliver all the interventions under the supervision of a clinical psychologist and a nursing specialist on the research team. Participants may be accompanied by family caregivers if necessary. To facilitate self-management, a brochure with summarized content of each session is to be given to each participant at the end of each session to bring home. Two home visits at 2 and 5 months after the intervention, respectively, are paid to each participant to answer any questions they have about practicing the skills at home and to consolidate their learning.

### Participants

We aim to recruit 150 participants from 20 social centers for older people and outpatient geriatric/pain clinics (i.e., clusters) in Hong Kong, with 6–12 participants per cluster. Efforts will be made to approach more service units until the target sample size is reached. Having four repeated measurements (i.e., baseline, posttreatment, and two follow-ups) and assuming the ratio of time-effect variance at the person level to the sum of random intercept variance and time-level residual variance is 0.30 (for yielding conservative power estimates), six participants per cluster per experimental condition are sufficient to detect a small to medium treatment × time interaction effect (Cohen’s *d* = 0.35) at α = 0.05 (two-tailed) and power of 0.80 [41, 42].

The inclusion criteria are (1) aged ≥ 60 years, (2) moderate chronic musculoskeletal pain operationalized as scoring ≥ 40 of 100 points on the pain intensity subscale of the Chronic Pain Grade questionnaire [43] in the past 3 months [44], (3) mild depressive symptoms as indicated by a score ≥ 4 on the ten-item Center for Epidemiologic Studies Depression Scale [45–47], (4) Cantonese speaking, and (5) having basic ability to read and write. Exclusion criteria are (1) insufficient fluency in Cantonese; (2) possible cognitive impairment as suggested by a score ≥ 3 on the Short Portable Mental Status Questionnaire (SPMSQ) [48]; (3) any impairment in basic activities of daily living as indicated by a score < 21 on a modified version of the Older Americans Resources and Services (OARS) Multidimensional Functional Assessment Questionnaire [49, 50]; and (4) physical conditions precluding participation in the intervention, including speech and hearing impairments. Research assistants introduce the study, participants’ rights, as well as potential risks, to each potential participant individually, from whom written informed consent is obtained.

### Randomization and blinding

Participants will be randomized by center/clinic, using a true random number generator, into either the multicomponent intervention or the pain education condition. The data collection site personnel and the participants will not be informed of the status of experimental assignment. The principle of restricted randomization will be applied to achieve a 1:1 ratio between the two treatment arms. The trainer who delivers the intervention will not be involved in outcome assessments, which are the responsibility of another research assistant blind to experimental assignment. Hence, this is a double-blind design.

### Measures

#### Mental status

The SPMSQ will be used to evaluate the mental functioning of the participants. The scale consists of ten items assessing participants’ memory, orientation, and mental operation for serial events. It has a maximum score of 10, with a score ≥ 3 suggesting at least mild cognitive impairment. The 4-week test-retest reliability was found to be 0.83 in the validation study [48].

#### Presence of chronic pain

Chronic pain will be determined by affirmative answers to both of the following questions: (1) “Are you currently troubled by physical pain, either all the time or on and off?” and (2) “Has this pain persisted for at least 3 months?” [51]. Subjects answering yes to both questions will then be asked to specify the duration of pain and the pain sites.

#### Pain intensity

Pain intensity will be measured by four instruments: (1) the pain intensity subscale of the Chronic Pain Grade questionnaire, (2) the visual analogue pain scale, (3) the Faces Pain Scale, and (4) the Verbal Rating Scale. The Chronic Pain Grade questionnaire pain intensity subscale has three items measuring pain intensity at present, at the worst time, and on average within the past 3 months on a scale of 0 (no pain) to 10 (pain as bad as could be). The original scoring averages the three items and multiplies it by 10 to yield a maximum of 100 [43]. The visual analogue pain scale is a continuous outcome measure consisting of a 10-cm line from 0 to 10 with low- and high-end points of “no pain” and “pain as bad as could be”; respondents are asked to put an “X” on the line to indicate their pain intensity in the preceding week [52, 53]. The Faces Pain Scale consists of six facial expressions to illustrate a spectrum of pain intensity in the past week and is scored 0 (no pain facial expression) to 5 (extremely pain facial expression) [54, 55]. Finally, the Verbal Rating Pain Scale asks respondents to rate their pain intensity by choosing from among six descriptors: 0 = no pain, 1 = mild pain, 2 = moderate pain, 3 = severe pain, 4 = very severe pain, and 5 = worst possible pain [56]. Besides the pain intensity subscale, all are single-item measures. In this study, the scores for the Faces Pain Scale and the Verbal Rating Pain Scale will be multiplied by 2 to obtain possible maximum scores of 10 for both instruments. For the primary outcome measure, we will form a composite measure of pain intensity by aggregating the scores of the six items from the four instruments described above (here, scores of the Chronic Pain Grade questionnaire pain intensity items are not multiplied by 10, so that all items of the composite measure have a maximum score of 10). In a pilot study of 694 Hong Kong older adults, this composite measure had a Cronbach’s α of 0.90.

#### Pain interference

The pain interference subscale of the Chronic Pain Grade questionnaire (α = 0.89 [57]) contains three items measuring the degree to which pain has interfered with daily activity, social activity, and work ability in the past 3 months on a scale of 0 (no interference) to 10 (pain interference as bad as could be). The pain interference score is calculated by averaging the scores of the three items and multiplying the average by 10 [43].

#### Pain persistence

The Chronic Pain Grade questionnaire has one item asking participants to recall the number of days they have been kept from conducting their usual activities in the past 3 months because of pain [43].

#### Pain self-efficacy

The Chronic Pain Self-Efficacy Scale consists of 22 items that are grouped into 3 subscales: self-efficacy for pain management (5 items), self-efficacy for physical function (9 items), and self-efficacy for coping with symptoms (8 items). The items are rated on a 10-point Likert scale from 10 to 100, with higher scores indicating higher self-efficacy. Alpha coefficients of 0.88, 0.87, and 0.90 were reported for the English versions of the pain management, physical function, and coping with symptoms subscales, respectively [58]. Because no Chinese version existed for this instrument, the items were translated into Chinese using a back-translation procedure and were found to have α coefficients of 0.78, 0.91, and 0.88, respectively, in a pilot sample of 694 older adults.

#### Catastrophizing cognitions

The Pain Catastrophizing Scale will be used to assess the catastrophizing thinking of the participants in relation to pain [59, 60]. The instrument contains 13 items measuring 3 dimensions of catastrophizing, namely rumination, magnification, and helplessness. Participants will be asked to reflect on past painful experiences and to indicate the extent to which they had each of the 13 thoughts when experiencing pain, on a 5-point scale from 0 (not at all) to 4 (all the time). In this study, the total score will be used. Cronbach’s α for the whole scale was found to be 0.91 in the aforementioned pilot sample.

#### Pain coping

Coping will be measured using a 16-item scale constructed by the research team on the basis of existing pain coping scales in the literature [61, 62]. Two items were constructed to measure each of the following eight coping strategies: guarding, resting, asking for assistance, relaxation, task persistence, exercise/stretching, seeking social support, and coping self-statement. Participants indicate the number of days in the previous week during which a certain coping strategy was used.

#### Depressive symptoms

Depressive symptoms will be assessed using a ten-item version of the Center for Epidemiologic Studies Depression Scale [45, 46], which has been translated into Chinese and validated in Chinese older adults [47]. The α coefficient was 0.82 in the aforementioned pilot sample.

#### Health-related quality of life

This construct will be measured using the Medical Outcomes Study Short Form Health Survey (12 items, version 2). The 12 questions are summed to produce a physical component score and a mental component score [63, 64]. The scale has been translated into Chinese and validated locally [65]. The α coefficients were 0.72 and 0.77 for the physical and mental subscales, respectively, in the pilot sample.

#### Hip and knee muscle strength

The participants’ hip and knee muscle strength will be measured using a back strength dynamometer. Participants are instructed to hold the handle of the dynamometer at midthigh position and exert force to pull up the chain against the dynamometer until the strength or pain threshold is reached. This procedure will be repeated three times, and the averaged readings across the three trials will be the measure of muscle strength.

#### Other information and covariates

Besides sociodemographic data, including age, sex, education, marital status, and employment status, we also asked participants to provide information on smoking, drinking, physical activity, self-perception of aging, basic and instrumental activities of daily living, and healthcare use associated with pain problems in the past 3 months. Basic and instrumental activities of daily living are each measured with seven items (scored 1 = dependent, 2 = needs assistance, 3 = independent) on a modified version of the OARS Multidimensional Functional Assessment Questionnaire [49, 50]. Self-perception of aging will be measured using the Attitude Toward Own Aging subscale of the Philadelphia Geriatric Center Morale Scale [66].

### Safety and clinical monitoring

The study involves a pain education program and a multicomponent intervention consisting of physical exercise and cognitive behavioral techniques. The training activities consist of lectures, discussions, paper-and-pencil exercises, and simple stretching exercises. Participation in the study is entirely voluntary. During the intervention, participants will recall experiences related to their pain; although such experiences might not be pleasant, their recall will unlikely induce psychological harm. In addition, when the participants perform exercise, they may experience tiredness and soreness in their muscles and joints and may have the potential risks of injuries such as fall and sprain. Such injuries are highly unlikely if the proper instructions are followed.

For clinical settings, oversight is provided by research team members who are also supervisors of the respective clinical units. Adverse events, if any, will be handled according to established clinical protocol, such as arranging proper medical treatment in the case of injury, and will be discussed with the principal investigator (STC). For social centers, oversight is provided by STC in consultation with other research team members, and adverse events will be handled in the same manner as in clinical settings. During the first year of the implementation of this project, only one adverse event was reported, in which a participant was injured mildly while being assessed for muscle strength during baseline screening and was quickly treated by the medical staff on site; this participant then withdrew his consent.

### Data management and confidentiality

The data will be kept by STC and accessible by research team members. A personal identification number will be created for each participant to match data from the same person across waves, whereas personally identifying information will be stored separately. All personally identifying information will be discarded as soon as data collection is over. The entire dataset, anonymized, will be kept for 10 years after the completion of the project and will be available for inspection by ethics committees or other researchers who have questions about our publications.

### Statistical analysis

Intention-to-treat analyses will be performed by mixed-effects regression with full information maximum likelihood estimation using Stata version 11.2 software (StataCorp, College Station, TX, USA), specifying four repeated measurements (level 1) nested within individuals (level 2) who are then nested within centers/clinics (level 3). Imputation of missing data is not needed, because the conditional distribution of missing data in the whole dataset is incorporated into the estimation of parameters in full information models [67]. Within-person covariance over time will be specified using an unstructured model. The intercepts and effect of time will be specified to vary randomly at the participant level, whereas the effect of treatment will be specified as fixed effects. Cohen’s *d*, calculated by taking the difference of the adjusted means between two comparison groups and dividing it by the pooled SD, will be used to estimate the effect size of treatment.

### Dissemination plan

Findings derived from the trial will be published in peer-reviewed journals and will be made available in summary form to frontline professionals in Hong Kong.

## Discussion

As populations age, there will be more and more older people with chronic musculoskeletal pain that cannot be managed by pharmacological treatment alone. Evidence-based nonpharmacological interventions are very much needed. Such interventions are preferably designed in such a way that they are deliverable by a range of healthcare workers in different settings to improve access to pain management services, especially in places such as Hong Kong, where specialist manpower is very tight or lacking. The interventions should also be implementable at home to facilitate self-management by older adults.

The present multicomponent intervention was designed according to these principles and considerations, on the basis of existing evidence and guidelines about the use of physical exercise and cognitive behavioral techniques for the management of pain. This intervention is being subjected to an evaluation against a pain education program in a double-blind, cluster-randomized controlled trial. Although this pain education program was developed on the basis of an existing brochure, it is, in theory, a more “active” and powerful intervention in the sense that it invites participants to question, explore, discuss, and interact rather than simply to receive information passively, such as when reading a brochure. The design also ensures that participants in the two treatment arms will have the same degree of treatment exposure (i.e., the number of sessions and their durations are the same), thus providing a rigorous assessment of the efficacy of the multicomponent intervention. Positive findings derived from this trial are expected to encourage society-wide adoption of this intervention program for the benefit of our aging communities.

## Trial status

Trial recruitment started in May 2016 and was estimated to last until November 2017 (protocol version 1, dated January 27, 2017). Other than elaborations of specific aspects of the protocol per requirements of different ethics committees, no substantive changes to the initial protocol have been made. As of 14 April 2017, we had recruited 136 participants.
