# Correction to: A multicomponent intervention for the management of chronic pain in older adults: study protocol for a randomized controlled trial

**DOI:** 10.1186/s13063-021-05780-x

**Published:** 2021-11-25

**Authors:** Sheung-Tak Cheng, Ka Long Chan, Rosanna W. L. Lau, Monique H. T. Mok, Phoon Ping Chen, Yu Fat Chow, Joanne W. Y. Chung, Alexander C. B. Law, Jenny S. W. Lee, Edward M. F. Leung, Cindy W. C. Tam

**Affiliations:** 1 Department of Health and Physical Education, The Education University of Hong Kong, 10 Lo Ping Road, Tai Po, New Territories Hong Kong; 2 Department of Clinical Psychology, Norwich Medical School, University of East Anglia, Norfolk, NR4 7TJ UK; 3 Department of Rehabilitation and Extended Care, Kowloon Hospital, 147A Argyle Street, Kowloon, Hong Kong; 4 Department of Anesthesiology & Operating Services, Alice Ho Miu Ling Nethersole Hospital, 11 Chuen On Road, Tai Po, New Territories Hong Kong; 5 Department of Anesthesiology & Operating Theatre Services, Queen Elizabeth Hospital, 30 Gascoigne Road, Kowloon, Hong Kong; 6 Department of Medicine and Geriatrics, Princess Margaret Hospital, 2-10 Princess Margaret Hospital Road, Lai Chi Kok, Kowloon, Hong Kong; 7 Department of Medicine, Alice Ho Miu Ling Nethersole Hospital, 11 Chuen On Road, Tai Po, New Territories Hong Kong; 8 Department of Medicine and Geriatrics, United Christian Hospital, 130 Hip Wo Street, Kwun Tong, Kowloon, Hong Kong; 9 Department of Psychiatry, North District Hospital, 9 Po Kin Road, Sheung Shui, New Territories Hong Kong

**Correction to: Trials 18, 528 (2017)**

**https://doi.org/10.1186/s13063-017-2270-3**

Following the publication of the original article [1], we were notified that that the last name of the third author was incorrect.

- Originally published name: Rosanna W. L. Lam
- Corrected name: Rosanna W. L. Lau

The original article has been corrected.
